# Oxytocin Exhibits Neuroprotective Effects on Hippocampal Cultures under Severe Oxygen–Glucose Deprivation Conditions

**DOI:** 10.3390/cimb46060371

**Published:** 2024-06-19

**Authors:** Mara Ioana Ionescu, Ioana-Florentina Grigoras, Rosana-Bristena Ionescu, Diana Maria Chitimus, Robert Mihai Haret, Bogdan Ianosi, Mihai Ceanga, Ana-Maria Zagrean

**Affiliations:** 1 Department of Functional Sciences, Division of Physiology II-Neuroscience, Carol Davila University of Medicine and Pharmacy, 050474 Bucharest, Romania; mara-ioana.iesanu@umfcd.ro (M.I.I.); ioana.grigoras@ndcn.ox.ac.uk (I.-F.G.); rbi24@cam.ac.uk (R.-B.I.); diana.chitimus@rez.umfcd.ro (D.M.C.); robertharet@gmail.com (R.M.H.); ianosi.bogdan@gmail.com (B.I.); 2 Wellcome Centre for Integrative Neuroimaging, Functional MRI of the Brain, Nuffield Department of Clinical Neurosciences, University of Oxford, Oxford OX3 9DU, UK; 3 Department of Clinical Neurosciences, University of Cambridge, Cambridge CB2 1TN, UK; 4 NIHR Biomedical Research Centre, University of Cambridge, Cambridge CB2 0QQ, UK; 5 Department of Ophthalmology, University Medical Center Gottingen, 37075 Gottingen, Germany; 6 Department of Neurology, Stroke Unit, Neuromed Campus, Kepler University Hospital, 4020 Linz, Austria; 7 Section of Translational Neuroimmunology, Department of Neurology, Jena University Hospital, 07747 Jena, Germany

**Keywords:** perinatal asphyxia, hypoxic-ischemic encephalopathy, oxytocin, oxygen–glucose deprivation, hippocampal cell cultures, GABA

## Abstract

Perinatal asphyxia (PA) and hypoxic-ischemic encephalopathy can result in severe, long-lasting neurological deficits. In vitro models, such as oxygen–glucose deprivation (OGD), are used experimentally to investigate neuronal response to metabolic stress. However, multiple variables can affect the severity level of OGD/PA and may confound any measured treatment effect. Oxytocin (OXT) has emerged as a potential neuroprotective agent against the deleterious effects of PA. Previous studies have demonstrated OXT’s potential to enhance neuronal survival in immature hippocampal cultures exposed to OGD, possibly by modulating gamma-aminobutyric acid-A receptor activity. Moreover, OXT’s precise impact on developing hippocampal neurons under different severities of OGD/PA remains uncertain. In this study, we investigated the effects of OXT (0.1 µM and 1 µM) on 7-day-old primary rat hippocampal cultures subjected to 2 h OGD/sham normoxic conditions. Cell culture viability was determined using the resazurin assay. Our results indicate that the efficacy of 1 µM OXT treatment varied according to the severity of the OGD-induced lesion, exhibiting a protective effect (*p* = 0.022) only when cellular viability dropped below 49.41% in non-treated OGD cultures compared to normoxic ones. Furthermore, administration of 0.1 µM OXT did not yield significant effects, irrespective of lesion severity (*p* > 0.05). These findings suggest that 1 µM OXT treatment during OGD confers neuroprotection exclusively in severe lesions in hippocampal neurons after 7 days in vitro. Further research is warranted to elucidate the mechanisms involved in OXT-mediated neuroprotection.

## 1. Introduction

During the peripartum phase, the fetus is at risk of perinatal asphyxia (PA), which is associated with elevated mortality and morbidity rates in infants. PA can culminate in severe systemic and neurological manifestations, with neonatal hypoxic-ischemic encephalopathy (HIE) notably impacting the vulnerable and hypoxia-prone developing brain [1]. Hence, there is a pressing necessity to find new therapeutic modalities for addressing this pathology.

Oxytocin (OXT) is a hypothalamic neurohormone that is secreted by the posterior pituitary gland and has an important role in promoting birth, lactation, and mother-infant bonding [2,3,4]. During pregnancy and labor, OXT is secreted in pulses, which increase in frequency, duration, and amplitude throughout labor, leading to a 3–4 times increase in the OXT plasma levels at birth [5]. Importantly, OXT crosses the placenta and the neonatal blood-brain barrier [6], reaching effective concentrations in the neonatal central nervous system during labor [7]. Intrapartum OXT exposure deficiency has been correlated with the extent of neonatal brain injury, suggesting that OXT is a promising therapeutic avenue in HIE [8]. Increasing evidence underscores the central role of the maternal neurohormone OXT in facilitating neuroprotection against brain damage induced by PA. OXT may protect fetal neurons through the regulation of microglial activation [9,10] and modulation of brain excitability, facilitating an excitatory-to-inhibitory gamma-aminobutyric acid (GABA) switch [6,11,12,13] during childbirth.

GABA exerts a pivotal role in the central nervous system, being the major inhibitory neurotransmitter in the adult mammalian brain [14]. GABA_A_ receptors (GABA_A_Rs) are chloride (Cl^−^) channels that hyperpolarize mature neurons, counterbalancing excitatory input [15]. However, during the fetal period, GABA elicits a depolarizing response as a consequence of the high intracellular Cl^−^ concentration expressed in immature neurons [16,17], transiently providing the major excitatory input [18,19].

The modulation of GABA-mediated inhibition is crucial for neuronal development and function, with intracellular Cl^−^ concentration playing an essential role [20]. During early development, the Na-K-Cl cotransporter 1 (NKCC1) predominates, leading to Cl^−^ accumulation and excitatory GABAergic signaling [21]. However, as the K-Cl cotransporter 2 (KCC2) expression increases before birth, there is a reduction in intracellular Cl^−^ levels, facilitating an inhibitory GABAergic switch in mature neurons [6,22]. OXT influences Cl^−^ homeostasis by stimulating KCC2 phosphorylation, enhancing its activity, and promoting Cl^−^ extrusion [23,24]. This modulation, mediated by OXT receptors (OXTRs), contributes to the GABA switch during neuronal development [6,23]. Nonetheless, OXT’s effects are temporally restricted, showing efficacy only during early postnatal stages [23]. Additionally, disruption of OXTR expression leads to disturbances in KCC2 upregulation and delays in the GABA switch, underscoring the significance of OXT in neuronal maturation and function [23].

In vitro experiments have extensively utilized oxygen–glucose deprivation (OGD) to study ischemic neuronal damage in both cell cultures and acute tissue slices [25]. OGD studies usually assess the cellular viability of the neurons immediately after the exposure or after a post-OGD reperfusion-like period to report the injury severity [26,27]. The post-OGD cellular viability results are difficult to compare between studies, given the variations in the exposure’s actual duration and hypoxia level and the use of different cell viability assays. Thus, it is important to carefully evaluate the severity of the lesion when interpreting findings from OGD studies.

We have previously reported that modulation of Cl^−^ transport by OXT has a neuroprotective effect when administered to early-stage hippocampal cultures at 2 days in vitro (DIV) during OGD [11], as well as perinatally in vivo [28]. However, OXT’s potential neuroprotective effect on neuronal cultures at later stages of maturation remains uncertain. As described above, immature and maturing neurons differ significantly in their intracellular Cl^−^ concentration and subsequent reaction to fast GABAergic neurotransmission, with OXT exerting its neuroprotective effect during the first 5 DIV [23]. Therefore, it stands to reason that OXT could have a different effect on cellular viability after OGD at DIV7 when compared to DIV2 neurons. This could be of translational significance since rodents at postnatal days 7–10 represent an equivalent developmental stage to term infants [29].

Thus, in the present study, we aim to investigate the effect of OXT at different concentrations on the viability of DIV7 rat hippocampal cell cultures subjected to OGD and reoxygenation and how this effect changes with lesion severity.

## 2. Materials and Methods

### 2.1. Primary Cultures of Rat Hippocampal Neurons

All animal procedures were carried out on Wistar rats, with the approval of the local ethics committee for animal research following the European Communities Council Directive 2010/63/EU on the protection of animals used for scientific purposes. Primary hippocampal cell cultures were prepared from postnatal day zero (P0) Wistar rat pups using a previously described protocol [11,30,31].

The hippocampi were isolated from the meninges and vascular plexuses under a dissecting microscope. Next, the dissociated cells were obtained by mechanical trituration and a 20 min incubation in papain (Worthington, 3.8 U/mL, Lakewood, CA, USA) for further enzymatic dissociation. The resulting cell suspension was kept in a culture medium (CM) containing Neurobasal-A (Gibco, Thermo Fischer Scientific, Waltham, MA, USA) supplemented with B-27 (Invitrogen, Cat. No. 17504044, Carlsbad, CA, USA), l-glutamine (HyClone, 0.5 mM, Washington, DC, USA), and antibiotic-antimycotic (Invitrogen, Cat. No. 15240062). The cells were then plated on poly-D-lysine-coated (Sigma-Aldrich, 70,000–150,000 kDa, 100 µg/mL, Burlington, MA, USA) 24-well plates at a cell density of 150,000 cells/well. Cultures were maintained at 37 °C in a 5% CO_2_, humidified atmosphere. Following a 5-day incubation period, half of the CM volume in each well (250 µL) was removed and replaced with 400 µL freshly prepared CM.

This protocol enables the cultivation of relatively pure hippocampal cultures characterized by a glial cell population of less than 5%, as documented in previous studies [32,33].

### 2.2. Exposure to Oxygen–Glucose Deprivation

OGD was performed at DIV7 as previously described [31,34]. We employed hippocampal cultures at DIV7 as this age corresponds to the developmental stage typically studied in rodent models of HIE [35,36]. These models traditionally use rodents aged between postnatal days 7 and 10, which approximate the developmental stage of term human infants, historically established through postmortem brain weight measurements [37].

**Figure 1:**
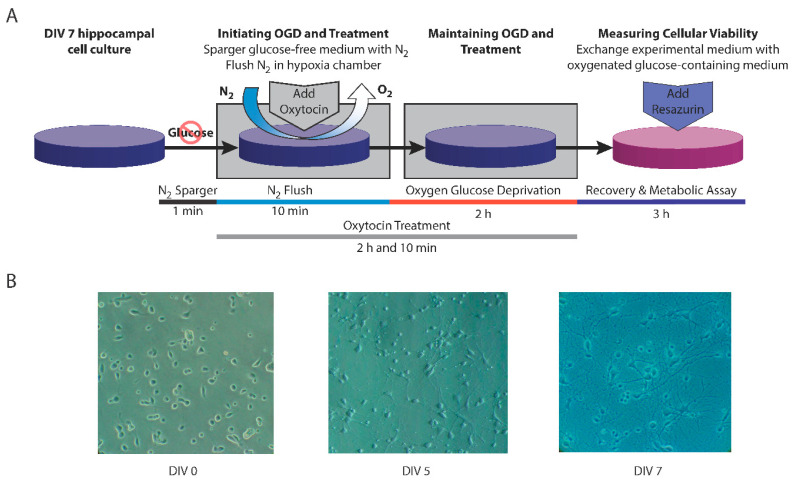
(**A**) Experimental design; (**B**) phase-contrast microscopy of cell cultures at DIV0, DIV5, and DIV7, 400× magnification. DIV—days in vitro; OGD—Oxygen–Glucose Deprivation; O_2_—oxygen; N_2_—nitrogen.

CM was removed from the wells and replaced with a deoxygenated experimental medium without glucose (EM-G), consisting of Neurobasal-A lacking both glucose and sodium pyruvate (Invitrogen), supplemented with HEPES (Sigma-Aldrich, 10 mM) and l-glutamine (HyClone, 0.5 mM). The plates were then promptly moved to a modular chamber (Billups-Rothenberg, San Diego, CA, USA), which was perfused with 100% N_2_ for 10 min, then hermetically sealed and kept in an incubator at 37 °C for 2 h. Control cultures were kept in an experimental medium consisting of Neurobasal-A with 25 mM glucose (EM + G) and sham normoxic conditions for 2 h. The experimental design is illustrated in Figure 1.

### 2.3. Treatment with Oxytocin

OXT treatments were applied during the 2 h exposure to OGD/sham normoxic conditions. Consistent with literature precedents, the DIV7 hippocampal cultures were treated with two different OXT concentrations previously employed in studies: 0.1 µM [23,38] and 1 µM [39,40]. Prior investigations have demonstrated a dose-dependent effect of OXT, with efficacy observed starting at 0.1 µM and reaching its peak at 1 µM [36].

### 2.4. Assessment of Cellular Metabolism and Viability

Post-exposure, the reoxygenated cell cultures were incubated at 37 °C and 5% CO_2_ for a 3-h time frame in a resazurin-containing medium to investigate their metabolic viability. Resazurin (Sigma-Aldrich) is a cell-permeable redox indicator that is irreversibly reduced in the mitochondria of viable cells to resorufin [11,31]. Thus, for the resazurin assay, EM-/+G was replaced with fresh assessment medium containing Neurobasal-A without phenol red (Gibco, Thermo Fischer Scientific), supplemented with HEPES (10 mM), l-glutamine (HyClone, 0.5 mM), and resazurin (Sigma-Aldrich, 100 µM). Resorufin fluorescence intensity was read at baseline and after a 3-h reoxygenation period at 535 nm excitation and 595 nm emission using a multimode detector (DTX880, Beckman Coulter, Brea, CA, USA).

### 2.5. Data Analysis

Data analysis and figures were rendered using GraphPad Prism 7.00 (GraphPad Software Inc. 9.3.0). The cellular viability for each condition was calculated as percentages compared to control values (normoxia + no treatment condition), considered 100%. The statistical unit was the number of culture plates tested per condition (n). All variables were tested for normality of distribution using the Shapiro–Wilk test, and central tendencies are reported as mean ± standard deviation (SD) unless otherwise stated. For normoxic data, statistical significance was evaluated using ordinary one-way ANOVA with a main factor of treatment (no treatment, 0.1 µM OXT, 1 µM OXT). For OGD data, statistical significance was evaluated using linear regression and paired t-tests. The significance threshold was set at *α* = 0.05.

## 3. Results

### 3.1. Oxytocin Did Not Affect the Viability of DIV7 Hippocampal Cell Cultures under Normoxic Conditions

The effect of OXT treatment on DIV7 hippocampal cell cultures under normoxic conditions was measured using ordinary one-way ANOVA with a main factor of treatment (no treatment, 0.1 µM OXT, 1 µM OXT). The effect of OXT treatment on the DIV7 cell cultures was not statistically significant (F(2, 23) = 1.289, *p* = 0.294; Table 1).

### 3.2. Cellular Viability after 2 h of OGD Followed a Normal Distribution

**Figure 2:**
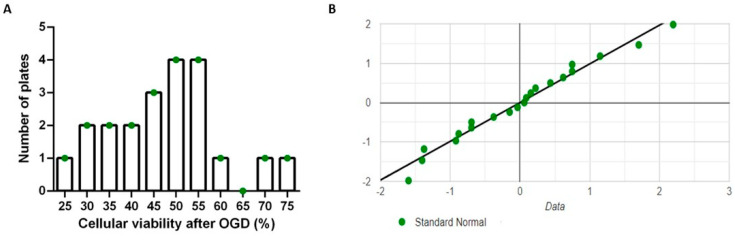
Cellular viability after 2 h of oxygen–glucose deprivation (OGD) follows a normal distribution. (**A**) Histogram of cellular viability (% of normoxic control condition) after 2 h of OGD exposure; (**B**) quantile–quantile plot.

The Shapiro–Wilk test was used to check for normal distribution of cellular viability following 2 h of OGD exposure with no treatment. The viabilities were normally distributed (*p* = 0.823, mean = 49.98%, SD = 12.72%, *n* = 21; Figure 2).

### 3.3. Oxytocin’s Effect on Cellular Viability Changed with OGD Severity

**Figure 3:**
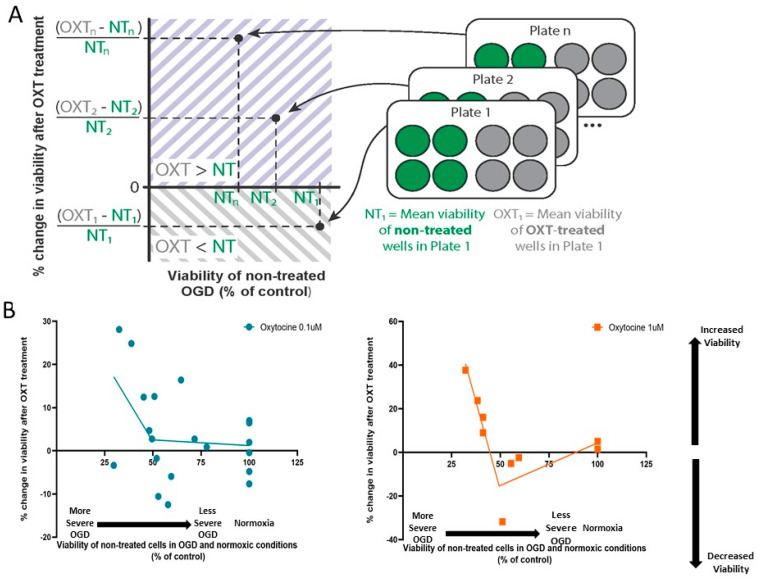
Oxytocin’s effect on cellular viability in relation to non-treated OGD viability, as measured by means of resazurin assay. (**A**) Schematic of data analysis and graphical representation of results. (**B**) Segmented regression of change in viability due to OXT treatment in non-treated cells in OGD and normoxic conditions: left—0.1 µM OXT, right—1 µM OXT. OGD—Oxygen–Glucose Deprivation; OXT—oxytocin.

For each plate exposed to OGD, we calculated the mean viability of non-treated OGD wells (NT-OGD) and the mean viability of the corresponding OXT-treated wells. We then calculated the percentage change in viability in OXT-treated wells compared to the non-treated ones as (OXT-NT)/NT. We can thus report plate-wise the effect of OXT treatment with respect to the OGD severity of the NT-wells. When cellular viability is higher in OXT-treated wells than in non-treated wells, this percentage change has a positive value, which would indicate neuroprotection; when cellular viability is lower in OXT-treated wells than in non-treated wells, this percentage change would have a negative value (Figure 3A).

We analyzed the relationship between the percentage of change in viability due to OXT treatment and OGD severity using linear regression and correlation analysis. The change in viability after 1 µM OXT treatment was negatively correlated with OGD severity (p_slope_ = 0.0393, R^2^ = 0.606, *n* = 7 culture plates with 38 wells). The change in viability after 0.1 µM OXT treatment was not correlated with OGD severity (p_slope_ = 0.206, R^2^ = 0.128, *n* = 14 culture plates with 41 wells).

The linear regression line intersected the abscissa at an OGD viability of 49.41%. Based on this breakpoint of 49.41% viability, segmented regression was further performed for both OXT concentrations in OGD and normoxic conditions (Figure 3B). For OGD viabilities <49.41%, 1 µM OXT was associated with increases in the viability of cells exposed to OGD, showing a neuroprotective effect. For OGD viabilities >49.41%, 1 µM OXT was not associated with a significant effect (Figure 3B). For OGD viabilities <49.41%, 0.1 µM OXT tends to increase the viability of cells, with no further impact for OGD viabilities >49.41% (Figure 3B).

### 3.4. Administration of 1 µM Oxytocin Increased the Viability of DIV7 Hippocampal Neurons Exposed to Severe OGD but Not of Those Exposed to Moderate OGD

As described above, we used linear regression to characterize the relationship between OXT-induced changes in viability and OGD severity. The linear regression line intersected the abscissa at an NT-OGD viability of 49.41%.

This cutoff was used to separate culture plates into two categories of OGD strength. Plates with mean viability of NT-OGD wells <49.41% were considered to have suffered a severe post-OGD lesion, while plates with mean viability of NT-OGD wells >49.41% were considered to have suffered a moderate post-OGD lesion.

**Figure 4:**
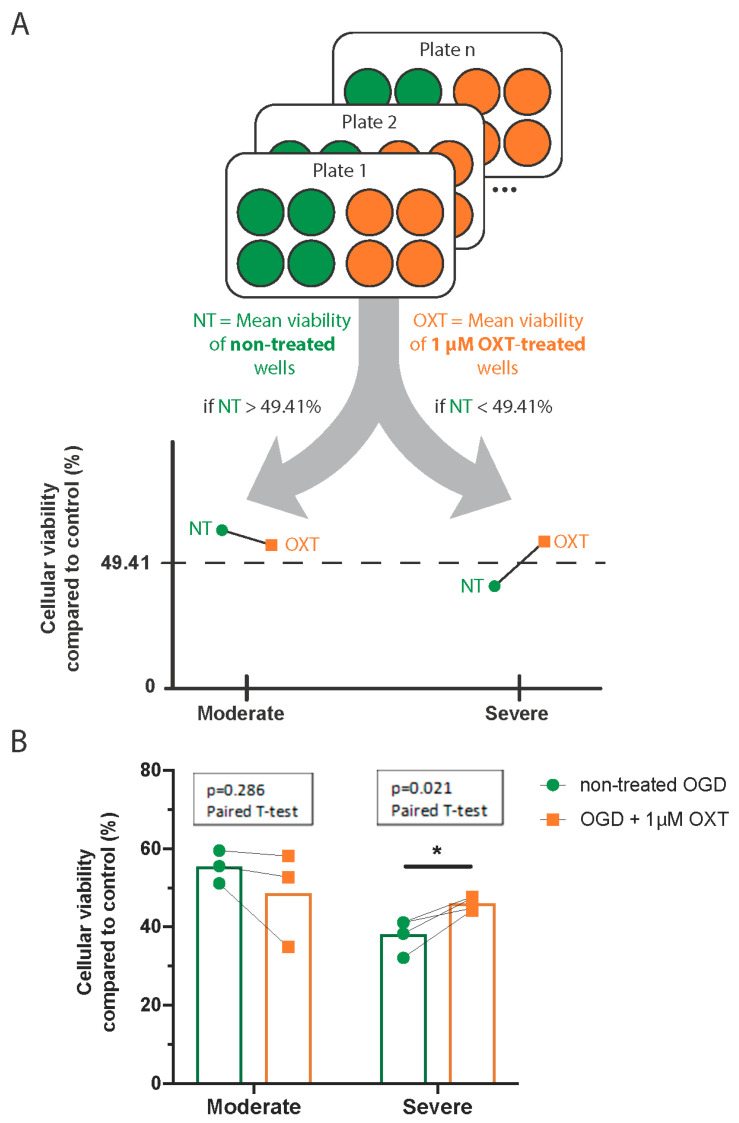
One µM of oxytocin increases the viability of cultures exposed to severe OGD but not of those exposed to moderate OGD. Schematic of data analysis and graphical representation of results (**A**). Viability of plates exposed to moderate and severe OGD, with or without OXT treatment (**B**). Individual data points are the mean cellular viability per culture plate. * *p* < 0.05. OGD—Oxygen–Glucose Deprivation; OXT—oxytocin.

For each plate exposed to OGD, we calculated the mean viability of NT-OGD wells and the mean viability of 1 µM OXT-treated wells (Figure 4A). The effect of 1 µM OXT treatment on DIV7 hippocampal cell cultures was compared to the viability of the corresponding NT-OGD using separate paired t-tests for moderate and severe OGD.

After a severe lesion, 1 µM OXT treatment significantly increased the viability of DIV7 hippocampal cell cultures (mean difference = 7.88%, 95% CI = [2.19; 13.57], *p* = 0.021). However, it had no effect on the viability of cultures exposed to moderate OGD (mean difference = 5.93%, 95% CI = [−8.99; 20.84], *p* = 0.286) (Figure 4B, Table 2).

### 3.5. Administration of 0.1 µM Oxytocin Had No Significant Effect on the Viability of Cultures Exposed to OGD, Regardless of OGD Severity

**Figure 5:**
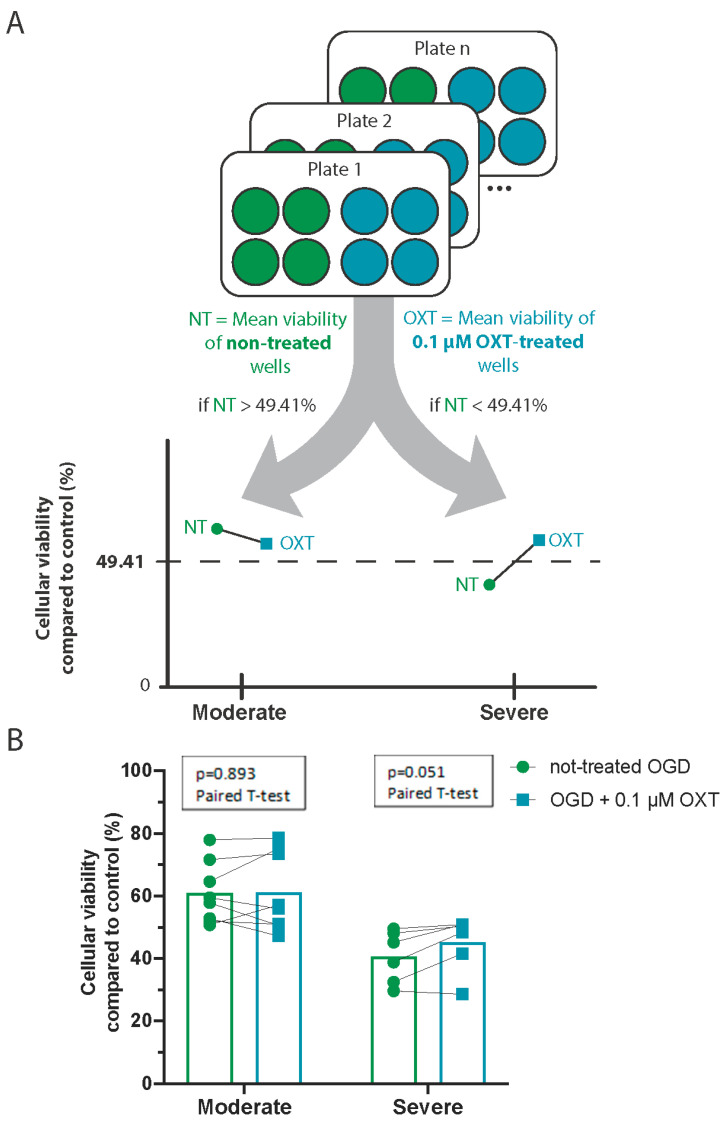
Oxytocin (0.1 µM) has no effect on the viability of cultures exposed to OGD, regardless of OGD severity. Schematic of data analysis and graphical representation of results (**A**). Viability of plates exposed to moderate and severe OGD, with or without OXT treatment (**B**). Individual data points are the mean cellular viability per culture plate. OXT—oxytocin; OGD—Oxygen–Glucose Deprivation; OXT—oxytocin.

For each plate exposed to OGD, we calculated the mean viability of NT-OGD wells and the mean viability of 0.1 µM OXT-treated wells (Figure 5A). The effect of 0.1 µM OXT treatment on DIV7 hippocampal cell cultures was compared to the viability of the corresponding NT-OGD using separate paired t-tests for moderate and severe OGD.

At 0.1 µM, OXT treatment did not significantly change the viability of DIV7 hippocampal cell cultures exposed to moderate OGD (mean difference = 0.30%, 95% CI = [−4.732; 5.324], *p* = 0.893). For severe OGD lesions, there was a trend towards neuroprotection, which failed to reach statistical significance (mean difference = 4.50%, 95% CI = [−0.054; 9.061], *p* = 0.051; Figure 5B, Table 3).

## 4. Discussion

Here, we investigated the effect of OXT on DIV7 hippocampal neurons following OGD and how this effect depends on OGD severity, considering that GABA_A_Rs have shifted to inhibitory effects in most neurons at this stage. The pathophysiology of brain ischemia is incompletely understood, aggravated in the developing brain by the high rate of simultaneous developmental changes. The energy deficit during ischemia influences the activity of ion pumps and results in electrolyte imbalances, which can, in turn, affect the activity of receptor channels, such as GABA_A_ channels [41].

In this study, we focused on hippocampal cultures due to the hippocampus’ vulnerability to hypoxia and its critical role in memory and learning, which are often impaired in individuals experiencing PA [42]. DIV7 hippocampal cultures, associated with a physiological excitatory-to-inhibitory shift in GABAergic signaling [43] and synapse formation [44,45], represent a specific developmental window [46]. Previous studies show that neurogenesis and synaptic integration in dissociated hippocampal cultures continue within 10 days of cells in vitro [46]. This underscores the relevance of our model, as the DIV7 cultures capture a critical period of early network formation and synaptic activity, aligning with the developmental stage of term infants [36]. Additionally, while DIV7 cultures exhibit single spikes and lower synaptic density compared to older stages, they still represent a fundamental phase of network maturation [47]. Moreover, there is evidence of a hyperpolarizing effect of GABA_A_R from both DIV6 primary rat neuronal cells and hippocampal slices from post-natal day 7 rats [36,39]. The preservation of this GABA switch across various brain structures and species, spanning from frogs, turtles, and rats to birds and likely extending to humans, indicates its evolutionary conservation and critical role in brain development [29,48].

OXT plays an important role in orchestrating the developmental switch from excitatory to inhibitory GABA signaling in the mammalian brain, but only within a limited temporal window during the peripartum period [6]. While prior research has highlighted the neuroprotective potential of OXT in immature hippocampal cell cultures [11], probably attributed to its facilitation of the physiological GABA switch, its impact on neurons exposed to OGD after this period remains uncertain. Notably, significant changes to Cl^−^ homeostasis have been reported to take place during the first week in vitro: KCC2 levels are reported to increase 15 times and NKCC1 to decrease four times at DIV6 compared to DIV1 [23], which is likely to impact the effect of OXT. Thus, this specific phase is crucial for studying OXT’s effects on neuronal connectivity and function. By focusing on this developmental stage, we provide valuable insights into OXT’s effects, which can be further validated in more complex in vivo models.

Our results showed that OXT treatment did not significantly impact the viability of DIV7 hippocampal cell cultures in control normoxic cultures. This is consistent with previous literature: when administered after DIV5 in normoxic conditions, OXT treatment did not lead to significant changes in chloride homeostasis in hippocampal neurons [23].

After 2-h OGD, we observed a wide range of values for mean cellular viability (between 25% and 75% of control viability), highlighting the variability in OGD vulnerability between cultures. This may be due to phenotypical differences in the pups or the method itself, including slight differences in hypoxia levels. Similar variability is seen in the wider OGD literature, perhaps due to non-standardized OGD and/or reperfusion durations, as well as different viability assays [25]. For example, cellular viability can range from 50% after a 30 min OGD [49] to approximately 60% after a 2 h OGD [50], with other studies reporting 52% viability after a 90 min OGD [51] and as many as 70% pyknotic nuclei reflecting apoptosis markers after a 4 h OGD exposure [52]. This variability may also reflect the real-life complexity of ischemic conditions where unpredictability is also an issue, emphasizing the need for careful consideration of sample size and standardized reporting of cellular viability after OGD exposure. Nevertheless, these simplistic models inadequately replicate the complex conditions observed in human scenarios.

Our study’s primary finding is that 1 µM OXT treatment during OGD had a significant neuroprotective effect only in hippocampal cultures severely affected by OGD, where neuron viability was less than 49.41%. In cultures with moderate lesions, where viability was above 49.41%, OXT did not exhibit a significant protective effect.

These findings may offer insights regarding previous conflicting reports using animals/neuronal cultures at different maturation stages and different severity lesions. Kaneko et al. reported that administering 1 µM OXT in DIV3 rat hippocampal cell cultures for 3 days before OGD led to a neuroprotective effect, but this effect was not observed if OXT was administered during OGD at DIV6 [39]. They reported approximately 70% cellular viability after OGD, a severity level categorized as moderate in our study, where we did not find significant neuroprotection. Furthermore, Kaneko et al. used cultures with 60% glial cells compared to our method’s <5% [53], suggesting that lesion severity may depend on the specific type of tissue or cells utilized. The neuroprotective effect of 1 µM OXT was also reported in an ischemia model consisting of exposure to OGD of hippocampal slices from 7 to 10-day-old rat pups, resembling hypoxic-ischemic lesions [36]. In this study, OXT mitigated neuronal death in a dose-dependent manner and delayed the anoxic depolarization in hippocampal CA1 neurons, likely by increasing GABA release through OXTR activation, enhancing the local inhibitory effect [36]. However, this study did not specify the precise viability of neurons.

Our findings are consistent with previous work showing that intranasal 1 µM OXT has a neuroprotective effect when administered to postnatal day-6 rat pups following an in vivo PA model [28]. Moreover, a clinical study linked the absence of intrapartum OXT exposure with the severity of neonatal brain injury, underscoring the OXT’s crucial role in severe PA outcomes [8].

Our current results align with reports suggesting that after OGD-induced ischemic lesions, neurons could return to a functionally immature phenotype, characterized by higher intracellular Cl^−^ concentrations and a subsequent decrease in GABAergic inhibition [54,55,56,57]. In both ischemic and traumatic injuries in the adult brain, GABA can return to a functionally immature phenotype [58,59]. This shift is driven by changes in the expression and activity of cation/chloride co-transporters. Specifically, KCC2 mRNA and protein levels are down-regulated within the first hours following ischemia [55,56,60], while NKCC1 activity increases through phosphorylation during 2 h reoxygenation [61]. This subsequently leads to an increased intracellular Cl^−^ concentration [57], which may result in reduced Cl^−^ influx through GABA_A_R and decreased GABAergic inhibition [55,62]. As the severity of the injury increases, KCC2 is more strongly downregulated [56], and GABAergic inhibition appears to decrease, being eventually suppressed in anoxic conditions [63]. The severity of the ischemic injury seems, therefore, to play an important role in the magnitude of GABAergic dysregulation. Moreover, given that these alterations occur within minutes to hours following the ischemic event, it is plausible that similar changes may also manifest in our model, which assessed 2 h OGD and 3 h reoxygenation.

OXT’s role in Cl^−^ homeostasis in immature neurons includes stimulating KCC2 phosphorylation and activity [39], and it might regain this effect in post-OGD functionally immature neuronal phenotypes. By enhancing KCC2 activity in these neurons, OXT would contribute to correcting the post-ischemic chloride imbalance and, therefore, exert a neuroprotective effect.

We report that 0.1 µM OXT did not have a statistically significant effect on DIV7 hippocampal cell cultures exposed to OGD, regardless of the severity of the lesion, though we could observe a trend towards neuroprotection in the severe lesions. Previous studies on immature neurons showed significant neuroprotective effects of 0.1 µM OXT on cell cultures after OGD exposure, as well as important effects on neuronal development and function [23,38]. This difference may arise from the type of neurons used in these studies. It is likely that, even though 0.1 µM OXT has significant effects on immature neurons, the concentration might be too low to elicit similar effects in neurons at this developmental stage since we could notice a trend towards neuroprotection.

These study’s findings are important for clinical practice as they suggest that a treatment effect may depend on the ischemic lesion’s severity. Therapeutic options in PA and subsequent HIE are currently limited, and translating the results from animal studies to clinical practice has proven extremely difficult. Despite advances in medical care, only hypothermia has shown neuroprotective benefits for term neonates with HIE after PA [64]. However, there is little evidence on the efficacy of hypothermia in reducing mortality and mitigating neurodevelopmental disability among infants with PA [65], creating a real need to develop treatment solutions for neuroprotection in PA. Furthermore, based on our present finding, it is advisable to categorize patients with PA according to the severity of their lesions, as some treatments may demonstrate efficacy specifically in cases of severe injury.

### Study Limitations and Future Perspectives

This study utilized in vitro hippocampal cell cultures, which do not fully recapitulate the complexity of the in vivo brain environment. Recent evidence indicates significant transcriptional disparities between primary hippocampal cultures and their in vivo tissue counterparts. Additionally, focusing solely on the hippocampus does not account for other brain areas affected by PA, where OXT is also known to influence neuronal activity.

The 2 h OGD model used here, while offering controlled conditions, does not fully mimic the multifaceted pathophysiology of PA observed clinically. The resazurin reduction method employed to assess cell metabolism has limitations, such as potential resazurin pool depletion with high numbers of viable cells and the generation of non-fluorescent hydroresorufin, which may lead to underestimating cell viability. We acknowledge the limitation of using a single viability assessment method. Despite inherent variability between cell cultures and OGD exposures, which could introduce confounding factors, our findings significantly advance the understanding of neuroprotection in OGD/PA-associated lesions.

Future research should include neurons from other brain regions to provide a comprehensive view of OXT’s neuroprotective effects and uncover additional protective mechanisms across various neural substrates. Incorporating cortical neurons, which are sensitive to hypoxia and modulated by OXT, could enhance the broader applicability of our findings. In vivo models are crucial to assess OXT’s effectiveness in an intact nervous system, addressing the limitations of our in vitro approach and exploring OXT delivery methods that minimize maternal impact. This research will bridge the gap between basic neuroscience and clinical applications, contributing to therapeutic strategies for neonatal care following PA.

## 5. Conclusions

In conclusion, this study provides insights into the neuroprotective role of OXT in the context of OGD as a model of HIE. We find that 1 µM OXT treatment during 2 h OGD exerts a neuroprotective effect on DIV7 hippocampal cell cultures only in case of a severe OGD-induced lesion, a condition which can be accompanied by a change in the neuronal phenotype into a functionally immature one. OXT treatment had no significant effect when administered during moderate OGD exposure or in normoxic conditions. Our results suggest that OXT might activate neuroprotective mechanisms in the developing neurons at DIV7 impacted by severe ischemia.

Our findings underscore the importance of carefully considering the developmental stage of neurons and the severity of the ischemic insult in assessing the efficacy of OXT treatment. Furthermore, our results from in vitro models may inform further in vivo research investigating the underlying mechanisms of OXT-mediated neuroprotection and clinically relevant studies optimizing therapeutic strategies for managing PA and subsequent HIE. Ultimately, the identification of OXT as a potential therapeutic target from in vitro studies holds promise for improving outcomes in neonates at risk of neurological injury due to PA, offering hope for novel interventions in clinical practice.

## Figures and Tables

**Table 1:** Mean cellular viability of DIV7 hippocampal neurons after OXT treatment under normoxic conditions.

	Mean Viability	SD	Number of Plates	Total Number of Wells	*p* Value Paired *t*-Test
Non-treated normoxia	100%	0	17	30	
0.1 µM OXT	101.0%	5.532%	7	28	0.605
1 µM OXT	104.3%	0.870%	2	7	0.341

DIV—days in vitro; SD—standard deviation; OXT—oxytocin.

**Table 2:** Mean cellular viability of DIV7 hippocampal neurons exposed to OGD with or without 1 µM OXT.

		Mean Cellular Viability	SD	Number of Plates	Total Number of Wells	*p* Value Paired *t*-Test
Moderate OGD	No treatment	55.39%	4.2%	3	19	
	1 µM OXT	48.58%	12.15%	3	15	0.286
Severe OGD	No treatment	38.17%	4.25%	4	21	
	1 µM OXT	46.05%	1.78%	4	23	0.021

DIV—days in vitro; SD—standard deviation; OXT—oxytocin; OGD—oxygen–glucose deprivation.

**Table 3:** Mean cellular viability of DIV7 hippocampal neurons exposed to OGD with or without 0.1 µM OXT.

		Mean Cellular Viability	SD	Number of Plates	Total Number of Wells	*p* Value Paired *t*-Test
Moderate OGD	No treatment	60.89%	9.85%	8	27	
	0.1 µM OXT	61.18%	12.57%	8	22	0.893
Severe OGD	No treatment	40.64%	8.31%	6	22	
	0.1 µM OXT	45.13%	8.8%	6	26	0.051

DIV—days in vitro; SD—standard deviation; OXT—oxytocin; OGD—oxygen–glucose deprivation.

## Data Availability

Data is contained within the article.
